# Temporal variation in daily temperature minima in coral reefs of Nanwan Bay, Southern Taiwan

**DOI:** 10.1038/s41598-020-65194-8

**Published:** 2020-05-26

**Authors:** I-Huan Lee, Tung-Yung Fan, Ke-Hsien Fu, Dong Shan Ko

**Affiliations:** 1 Department of Oceanography, National Sun Yet-Sen University, Kaohsiung, Taiwan, ROC; 2 National Museum of Marine Biology and Aquarium, Pingtung, Taiwan, ROC; 3 Institute of Marine Biology, National Dong Hwa University, Pingtung, Taiwan, ROC; 4 Marine Science and Information Research Center, National Academy of Marine Research, Ocean Affairs Council, Kaohsiung, Taiwan, ROC; 5 Oceanography Division, Naval Research Laboratory, Stennis Space Center, Mississippi, USA

**Keywords:** Marine biology, Physical oceanography

## Abstract

Temporal variation in seawater temperature plays a crucial role in coral reef ecology. Nanwan Bay, Southern Taiwan is home to well-developed coral reefs, which frequently experience cold-water intrusions caused by internal wave-induced upwelling, that manifest in distinct daily temperature minima. These temperature minima and their associated sources were studied by recording *in situ* bottom temperatures and sea levels observed at depths of 5 and 30 m from May 2007 to September 2008. These data were then compared to the East Asian Seas Nowcast/Forecast System, and it was found that daily temperature minima presented large variations with magnitudes of 2–3 °C over periods from days to months. It was further demonstrated that the cold-water intrusions may have originated from depths of ~100 m and were strongly affected by westward propagating mesoscale eddies from the Pacific basin. An impinging warm anticyclonic eddy in July 2007 may have combined with the El Niño, resulting in temperatures surpassing 29 °C and degree heating days >4.0 °C-days at both depths, which were coincidental with a mass coral bleaching event. This eddy’s impact was additionally evident in high correlations between daily temperature minima and residual sea levels, suggesting that mesoscale eddies alter stratification, substantially influence temperature variation, and play important roles in understanding ecological processes on coral reefs.

## Introduction

Marine environmental conditions vary on multiple temporal scales, and under the influence of climate change, these conditions are likely to change in frequency and magnitude. Seawater temperature is a crucial environmental factor in determining drivers of coral physiology and coral reef ecology^1–4^. Mass coral bleaching and mortality, caused predominately by abnormally warm events^5–7^ and sometimes by anomalously cold ones^8,9^, highlight the importance of understanding and forecasting seawater temperature variations and extremes^10–12^.

The effect of seasonal and monthly (lunar) variations in seawater temperature on coral reef ecosystems is well recognized^13^, yet temporal variation on a daily to weekly or shorter scales is rarely studied^7^. This is unfortunate given that corals exposed to large daily temperature fluctuations may possess elevated thermal tolerance^12,14^. The risk of coral bleaching may also be reduced by high-frequency temperature variability^12^. The largest temperature fluctuations in coral reefs are often associated with intermittent upwelling induced by internal waves^7,12,13^. Since upwelled cold water has the capacity to act as a buffer from thermal stress during abnormally warm events, these reefs may become refuges in a warming ocean^14–17^. Corals inhabiting intermittent upwelling environments have shown a variety of benefits, ranging from improved growth^18^ and energy reserves^19^, to enhanced metabolic plasticity^20^, and site-specific physiological acclimatization^21,22^. Yet, the influence of mesoscale eddies on corals in these dynamic environments remains unclear^23,24^.

**Figure 1:**
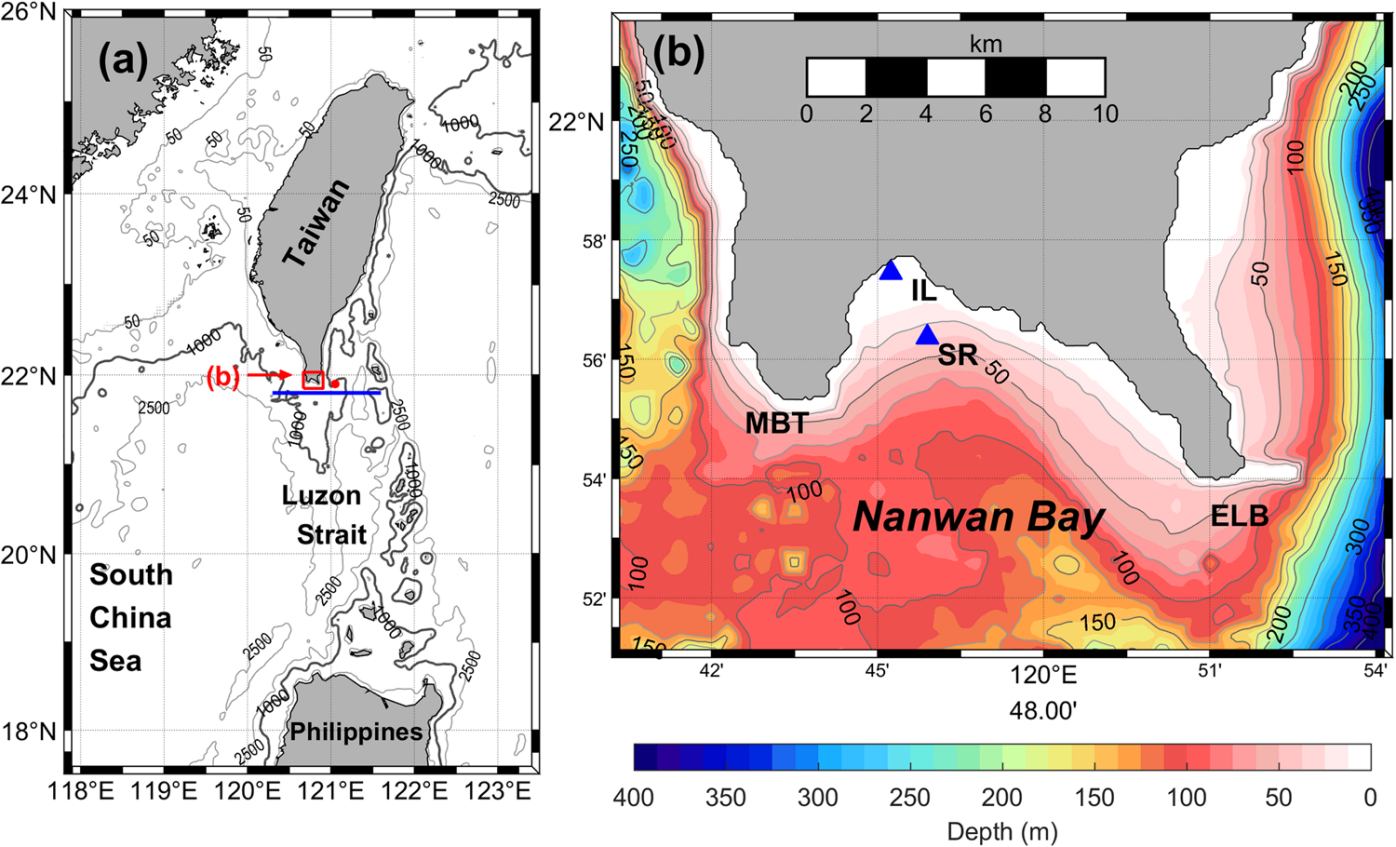
Bathymetry of Nanwan (NW). (**a**) Large-scale view of study sites; specific details for NW (red box) are shown in (**b**). The blue line and the red dot are positions used for model data collection (see Figs. 5 and 6); (**b**) A detailed map of NW. “▲” denote the temperature and sea level station at IL and the temperature station at SR. The contour interval (grey) is 25 m. Eluanbi (ELB) and Maobitou (MBT) are the east and west boundaries of NW, respectively.

Fringing coral reefs are well-developed along the coast and down to ~30 m depth in Nanwan Bay (NW), a small semi-enclosed bay approximately 15 km wide, at the southern tip of Taiwan (Fig. 1). The topography of NW is canyon-like, oriented in a northwest to southeast direction, with a maximum depth of ~80 m. Environmental conditions in this region show seasonal, lunar, and tidal variations due to several oceanographic and weather-related factors, including the influence of the South China Sea (SCS), Kuroshio current, tides, internal wave-induced upwelling, monsoons, and typhoons^4,13,25–27^. NW is predominately influenced by the SCS, which has a comparatively shallow thermocline and typically cooler temperature than the Kuroshio current^26^. The Kuroshio current typically flows along the east coast of Taiwan, although its path shifts and traverses the Luzon Strait in winter. The Kuroshio current’s path fluctuates frequently over weeks to months due to impinging mesoscale eddies. Some eddy-Kuroshio interactions east of Taiwan can strongly affect the Luzon Strait, for example, by forming a large anticyclonic “loop current”^27,28^. The largest known oceanic internal waves globally are generated within the ridges of the Luzon Strait south of NW^29^. Despite these locally unique phenomena, the role that mesoscale eddies play in influencing temperatures in NW is unknown.

NW is subject to significant cold-water intrusions, coinciding with tidal frequencies, with daily temperature differences exceeding 5 °C^25,30^. Several unusual biophysical phenomena associated with these temperature fluctuations have been reported. Three particularly unique examples include: (1) an unusual cold event in 1988, which decreased the sea surface temperature (SST) from ~25 °C to ~14 °C over the course of just a few hours and resulted in mass mortality of fishes^31^; (2) corals inhabiting a reef exposed to constant warm-water effluent from a nuclear power plant that have been able to not only survive, but thrive, in temperatures beyond their typical bleaching threshold (~29 °C)^32^, likely due to intermittent cold-water intrusion driven by internal waves induced upwelling^33^; and (3) the lunar timing of larval release of two brooding reef corals, which show temperature-related plasticity^2,4^. Regarding the latter, the peak reproductive timing shifted from around the full moon and spring tide in winter to around the first quarter moon and neap tide in summer; this may allow larvae released in the summer to avoid the impact of internal wave induced upwelling, which is strongest during the spring tide (full moon)^4^. In contrast, severe mass coral bleaching events occurred due to abnormally warm seawater temperatures during El Niño events in 1998, 2007, and 2016–17^34,35^.

Mechanisms for cold-water intrusions, including both local and remotely forced internal waves, have been explored in model-based studies^25,26,36^. However, the temperature variation in deep reefs, as well as the factors influencing the cold water intrusion in NW, has not been clearly addressed. A thorough assessment of *in situ* temperature data acquired at frequent (minutes-hours) intervals and assessed over long-term (weeks-years) timescales may help to shed light on these phenomena. Therefore, *in situ* bottom temperature measurements at depths of 5 and 30 m were recorded from 2007 to 2008. Complementary to these data, we also examined remote temperature data sets from the East Asian Seas Nowcast/Forecast System (EASNFS), developed by the US Naval Research Laboratory (NRL)^36,37^ to comprehensively describe the hydrographic field of NW. Our objectives were to (1) examine the temporal variation in temperature, with a particular emphasis on the minima, at shallow and deep coral reefs; (2) evaluate the possible roles of mesoscale eddies in influencing temperature patterns; (3) explore the possible sources of cold-water intrusions; and (4) examine the relationship between temperature and sea level in order to forecast temperature variation and extremes in NW.

## Results

**Figure 2:**
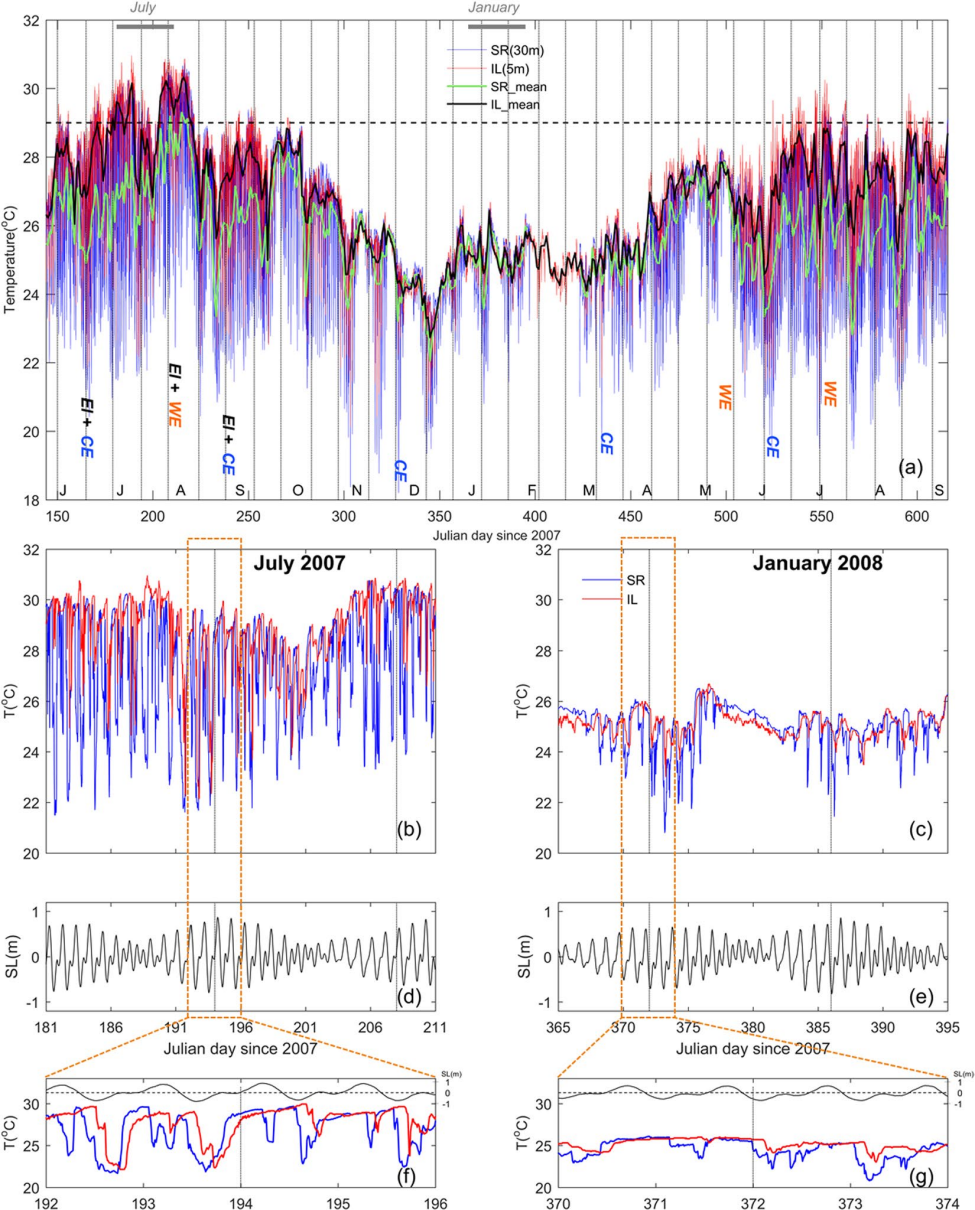
Time series of temperature (T) and sea level (SL) at S-Rock (SR) and the inlet (IL) of the third nuclear power plant. (**a**) Blue and red lines are observed raw data (temporal resolution of 10 min) for SR and IL, respectively. Green (SRmean) and black (ILmean) lines are the daily means. Vertical dotted lines mark the dates of the new and full moons. Capital letters at the bottom mark the first day of each month. The black, horizontal dashed line indicates the local coral bleaching threshold temperature of 29 °C. El (El Niño), CE (cold eddy), and WE (warm eddy) were derived from Fig. 7. Monthly and daily temperature variation for (**b,f**) July 2007 and (**c,g**) January 2008, as well as their corresponding sea level variations (**d**,**e**), respectively, are also presented. Vertical dashed lines mark the dates of the  new and full moons.

The observed raw temperatures at the bottom of a seamount, S-rock (SR), and at the inlet (IL) of the Tai-Power third nuclear power plant (Fig. 1) from May 2007 to September 2008 are shown in Fig. 2a, and a finer-resolution comparison of a representative summer (July) and winter (January) months is shown in Fig. 2b,c. Seasonal variation is clear, whereby temperature was warmer in the summer (June to October) and cooler in the winter (November to May). Strong temperature fluctuations (>5 °C) were recorded in summer; temperatures varied between 18.2 and 30.8 °C at SR and 20.1 and 31 °C at IL. The majority of daily temperature minima coincided with the spring tides, as indicated in the Fig. 2a by the dates of the new and full moons. In summer, the daily magnitudes were 4–6 °C (5–9 °C) and 2–5 °C (3–8 °C) during neap (spring) tides for SR and IL, respectively (Fig. 2d,f). During winter, daily fluctuations only occurred during the spring tides, with daily magnitudes of 2–4 °C and 1–2 °C for SR and IL, respectively (Fig. 2e,g).

**Figure 3:**
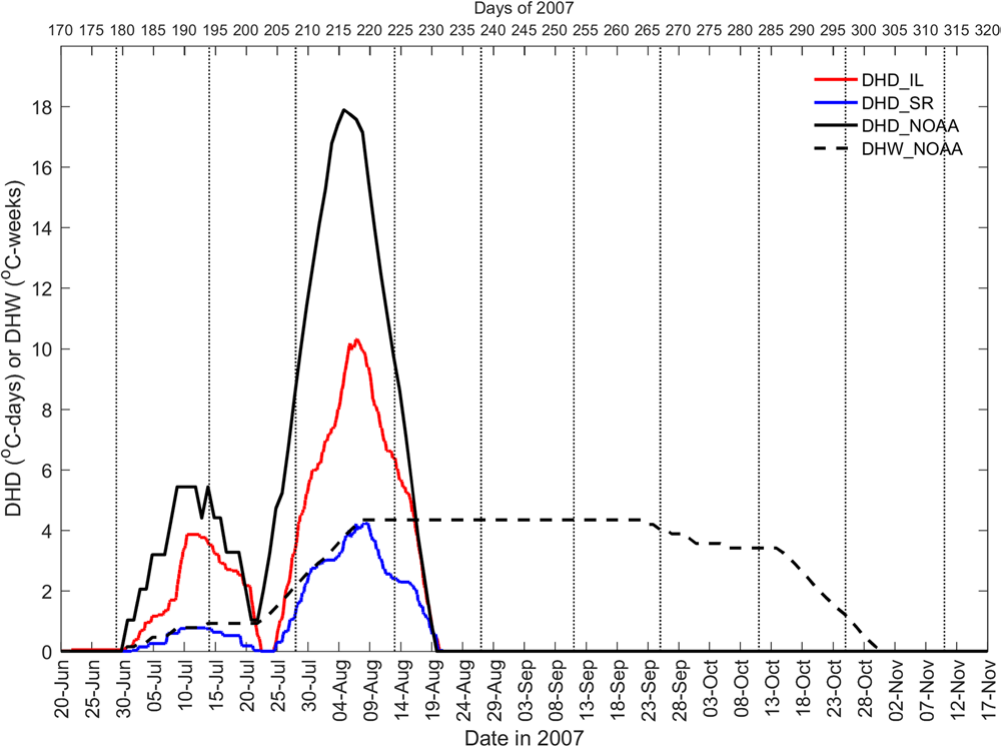
Time series of degree heating days (DHD) at SR and IL, as well as DHD and degree heating weeks (DHW) from NOAA satellite data. Vertical dotted lines mark the dates of the new and full moons.

In the summer of 2007, both the SRmean and ILmean exceed 29 °C, the local coral bleaching threshold^32^, for ~5 days (day 214–218) and ~17 days (day 203–219) during late July and early August, respectively (Fig. 2a). The degree heating day (DHD)^7^ peaked at 10.31 °C-days on 7 August and at 4.25 °C-days on 9 August for IL and SR, respectively (Fig. 3). The DHD and degree heating week (DHW) from the United States National Oceanic and Atmospheric Administration (NOAA) Coral Reef Watch (CRW) satellite sensor data^38^ peaked at 17.89 °C-days on 4 August and at 4.34 °C-weeks during 8 August to 22 September, respectively. The SRmean and ILmean dropped and was lower than 29 °C due to the influence of three typhoons during 7 to 19 August (Fig. 2a).

**Figure 4:**
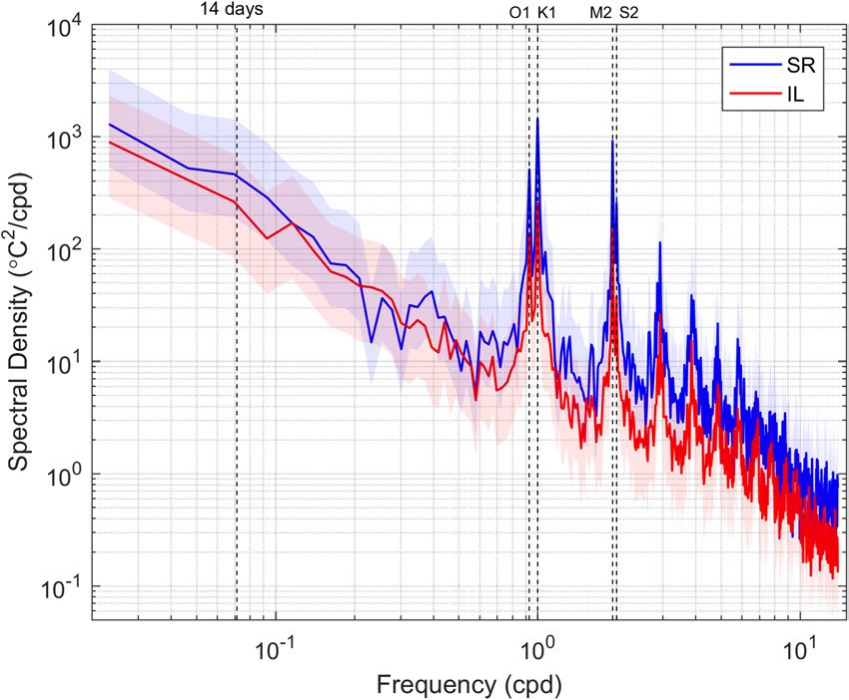
Spectra of temperatures. Blue and red lines represent SR and IL, respectively. Color shadings represent the 95% confidence interval. Cpd = cycle per day.

The predominance of tidal fluctuations can be clearly seen in the corresponding temperature spectra (Fig. 4). Both records showed distinct energy peaks at the diurnal (~24 h [K1 and O1]), semidiurnal (~12.5 h [M2 and S2]), mixed (8 h), and higher tidal harmonics, as well as a smooth peak each fortnight related to spring-neap tides; energy peaks at SR were considerably larger than IL. Higher tidal harmonics might be caused by topographic effects since IL presented more peaks at higher frequencies than SR. The stronger signal at SR was likely related to larger temperature fluctuations since the magnitudes at SR were larger than IL. However, longer periods of motions cannot be well resolved because of data length limitations (i.e., periods depicting variation over longer-than-fortnightly scales are not evident).

The daily means of temperatures for SR (SRmean) and IL (ILmean) are shown in Fig. 2a, and both locations displayed similar seasonal patterns; SR varied between 22.1 and 29.3 °C and IL varied between 22.7 and 30.3 °C. SR was ~1–2 °C cooler during summer, but the two sites were approximately the same in winter. The annual temperature record showed large variations. Specifically, in summer 2007, the SRmean was ~26 °C in the middle of June (day 168), then increased to ~29 °C in early August (day 215), but decreased sharply to ~23 °C in the middle August (day 233). Over this same time period, the ILmean exceeded 29 °C in late June (day 171), and exceeded 30 °C in early July (day 188) and again in late July (day 205). In contrast, the 2008 summer averages (days 550–580) were considerably cooler; ILmean and SRmean were <29 °C and <28 °C, respectively.

**Figure 5:**
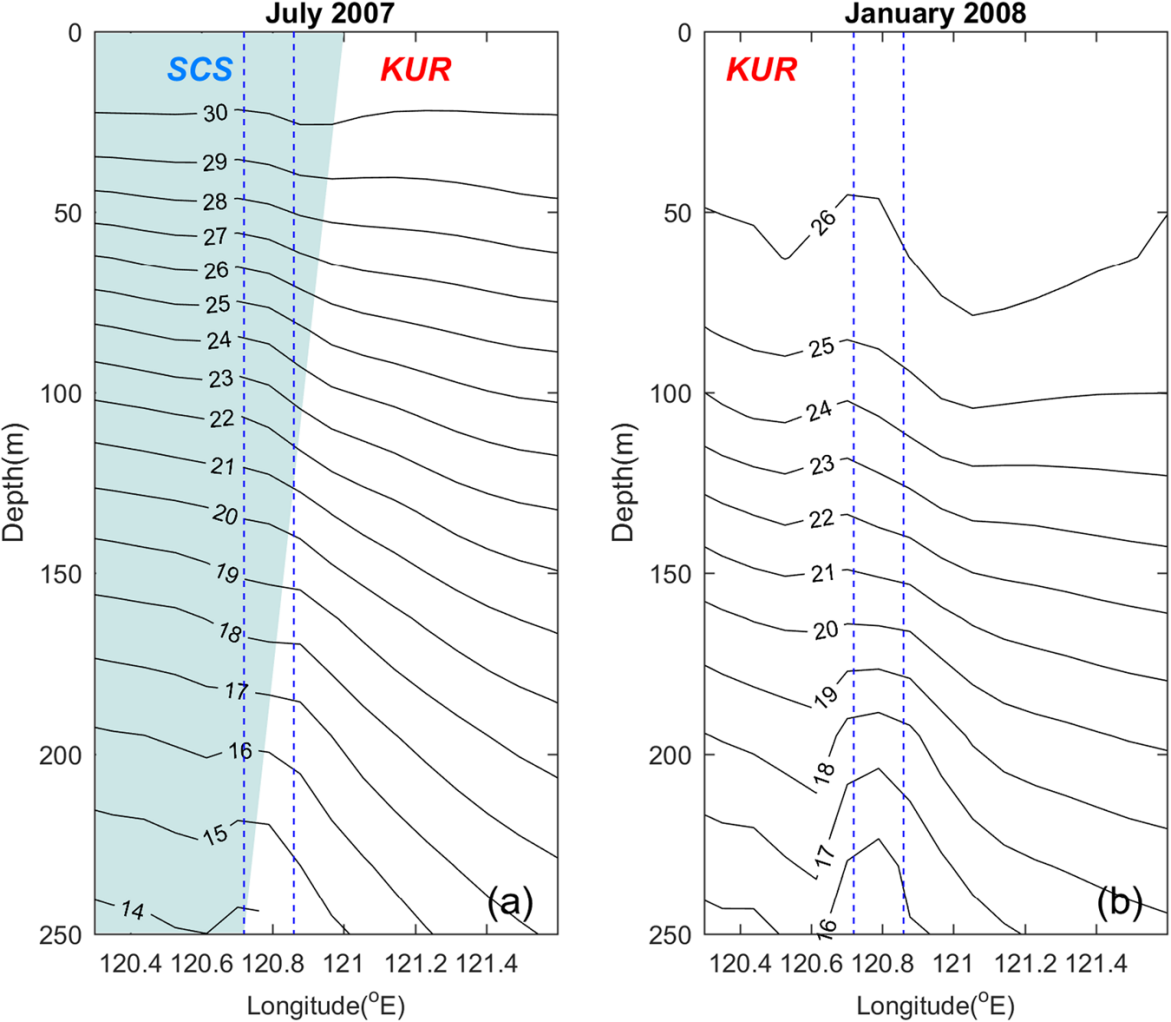
Monthly mean temperatures from the EASNFS model reanalysis at 21.8°N, 120.3–121.6°E (the blue line in Fig. 1a) for (**a**) July 2007 and (**b)** January 2008. The two blue dashed lines indicate the east and west boundaries of NW. South China Sea = SCS (shading) and Kuroshio current = KUR (white).

The magnitudes of the daily temperature fluctuations were greater in summer than in winter, suggesting changes in the vertical temperature gradient of the surrounding water. Unfortunately, there were no concurrent temperature measurements in the open ocean. We therefore used model temperatures for the study period from the EASNFS reanalysis which assimilates all available satellite SST, SSHA (sea surface height anomaly), and *in situ* hydrographic observations. Figure 5 shows zonal temperature sections in the upper 250 m from EASNFS at 21.8°N, 120.3–121.6°E (marked in Fig. 1a) for the representative summer (July 2007) and winter (January 2008) months.

In the summer, model temperatures decreased from 30 °C at the surface to 14 °C at 250 m depth, showing strong stratification (Fig. 5a). Zonal horizontal temperature gradients were clearly observed below 30 m: temperatures were colder in the South China Sea (SCS), whereby, the zonal gradient increased with depth. The steep zonal slope of the isotherms is indicative of the Kuroshio current. In winter, the upper layer (<100 m) became well-mixed, and the zonal temperature gradient was weak (Fig. 5b). The Kuroshio current also migrated westward into the SCS. The water mass around NW was replaced by the Kuroshio current. Stratification was strong during summer and caused large temperature fluctuations in NW, whereas the stratification was too weak during winter to sustain large temperature fluctuations.

**Figure 6:**
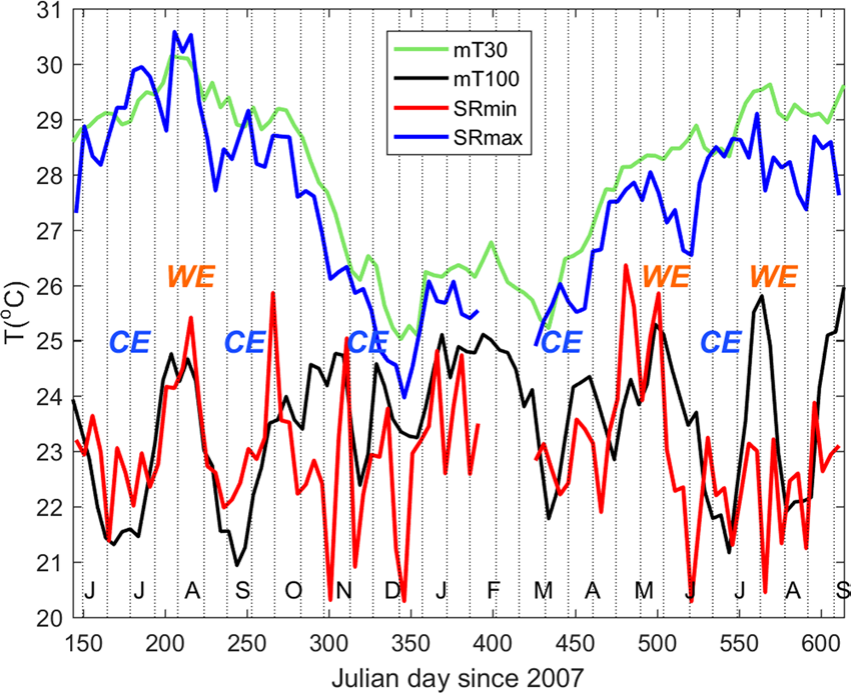
Time series of daily temperature (T) maxima and minima displayed as a 5-day average at SR (SRmax and SRmin, respectively), and EASNFS temperature output at 21.9°N, 121.054°E (the red dot in Fig. 1a) from depths of 30 m (mT30) and 100 m (mT100). CE (cold eddy) and WE (warm eddy) were derived from Fig. 7. Vertical dotted lines mark the dates of the new and full moons.

The lowest SR minimum temperature was 18.2 °C. Based on the model reanalysis, the cold water may have originated from depths shallower than 200 m (Fig. 5). We selected model temperatures at two depths to compare with our observations: 30 m, which is representative of the surface (mixed) layer, and 100 m, where the mean model daily temperature (~23.5 °C) was comparable to the mean observed daily temperature minimum (~23.0 °C) at SR. The chosen model grid was located at 21.902°N, 121.054°E, ~20 km east of NW at a depth of 1000 m (red dot in Fig. 1a). This location aimed to represent the surrounding conditions of the western North Pacific Ocean without interference from the coastal waters of Taiwan. Temperatures at the two depths (mT30 and mT100) were compared with the daily temperature maxima and minima at SR (SRmax and SRmin, Fig. 6). The daily maxima and minima at IL varied similarly but were warmer than SR. Correlation coefficients of SRmax vs. ILmax and SRmin vs. ILmin were 0.98 and 0.76, respectively.

The observed daily temperature maxima and minima were averaged into the same time interval as the model output (5-day mean). There were generally good agreements between SRmax and mT30 and between SRmin and mT100 (Fig. 6). The correlation coefficients for (1) SRmax and mT30 and (2) SRmin and mT100 were 0.92 and 0.56, respectively, with data from days 250–391 and 556–611 were discarded due to apparent differences; additionally, there were no data for days 392–425. This suggests that the model was able to capture background, low-frequency temperature fluctuations. SRmax and mT30 displayed seasonal variations: reaching ~30 °C in summer 2007, decreasing to ~25 °C in winter, then increasing to ~29 °C in summer 2008. The SRmax was generally colder by ~1 °C compared to mT30. This result indicates that, absent of cold-water intrusions, SR temperature varied with the surrounding ocean surface water.

Unlike SRmax, SRmin did not show seasonal variations (Fig. 6). Rather, SRmin was dominated by several large fluctuations with magnitudes of 3 to 5 °C in periods of weeks to months (e.g., the warm anomaly from the middle June to late August 2007 [days 165–240] noted earlier). A similar trend found between SRmin and mT100 indicated that the intruded cold water likely came from the open ocean at depths of ~100 m. SRmin, in addition, showed several sharp peaks (e.g., days 165, 180, 301, 316, 346 etc.) that were coincident with the full and new moons. The spring/neap modulation in SRmin likely reflects the large isotherm excursions in the Luzon Strait associated with strong internal waves. The internal wave component, however, was not included in the available EASNFS output.

**Figure 7:**
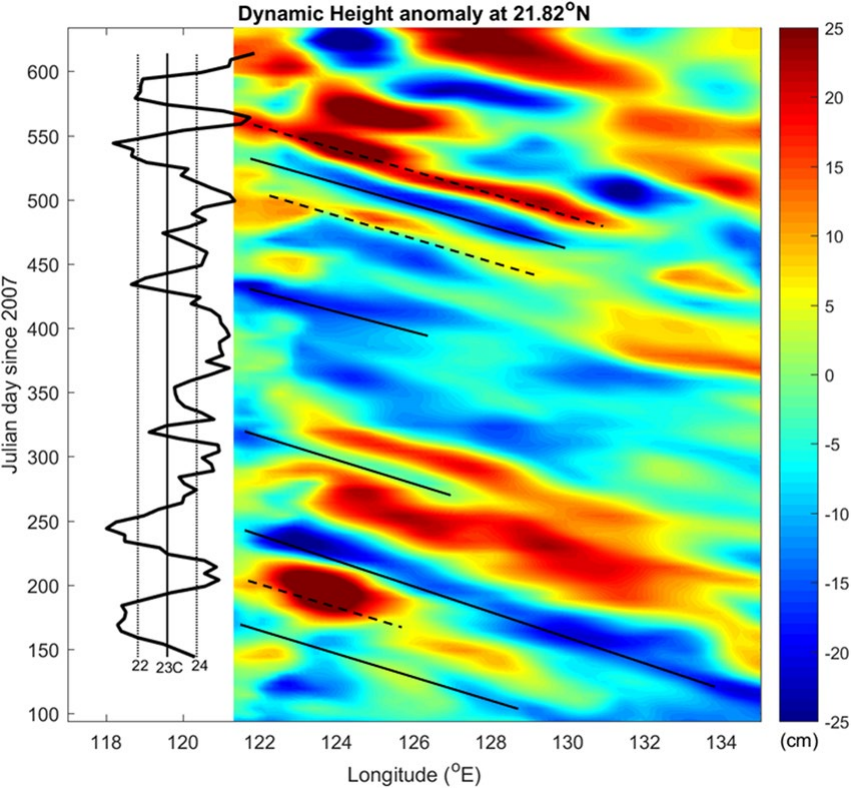
Time series of model dynamic height anomalies along 21.82°N with mT100 (black line) shown at the left of the plot. The solid/dashed lines mark the westward propagating cyclonic/anticyclonic eddies related to cold/warm anomalies of mT100.

Figure 7 shows the westward propagation of mesoscale eddies, as evidenced from the model dynamic height anomalies (referenced to 500 m) along 21.82°N. To relate NW temperature fluctuations to the approaching mesoscale eddies, mT100 data are displayed on the left in the same plot. The result shows that mT100 temperatures were warmer/colder when anticyclonic/cyclonic eddies appeared at 122°E. SRmin displayed similar behavior with mT100, as noted earlier (Fig. 6).

**Figure 8:**
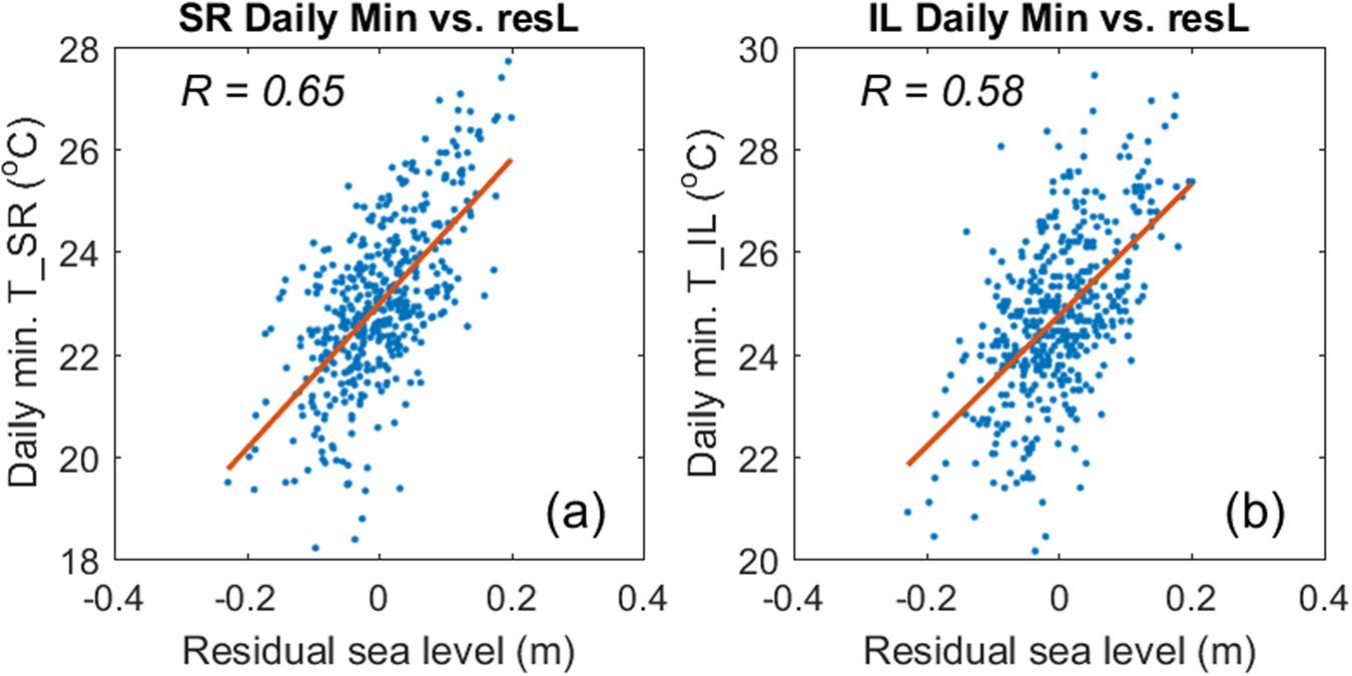
Scatterplots of daily residual sea levels (resL) vs. daily temperature minima of SR and IL. (**a**) resL vs. SRmin; (**b**) resL vs. ILmin. R = correlation coefficient.

The residual (detided) sea level variations (resL), calculated from the observed sea level variations at IL, with diurnal (K1, O1) and semi-diurnal (M2, S2) tides removed, were caused by both local and remote effects; the latter was expected to be related to the open-ocean dynamic heights, and hence mT100. Since mT100 and SRmin were related, it was of interest to check if resL was correlated to SRmin and ILmin. Figure 8 shows the scatterplots of resL vs. SRmin and resL vs. ILmin. Both SRmin and ILmin were correlated to resL, with correlation coefficients of 0.65 and 0.58, respectively. This supports the hypothesis that the residual sea level variations in NW are mainly controlled by impinging mesoscale eddies.

## Discussion

Although temporal variation in seawater temperature in shallow (5–10 m) coral reefs has been well studied in NW^4,13,25,26,31–33^, this study is the first to examine the temporal variation in seawater temperature at a deep (30 m) coral reef in NW. The daily temperature minima showed large magnitude fluctuations (2–8 °C) over periods from weeks to months (Figs. 2, 6), and this week-to-month-scale variation at 30 m is in contrast to the scale and magnitude of temporal variation occurring at other deep coral reefs globally: e.g., Conch Reef, Florida Keys (45 m)^39^, Curacao, Netherlands Antilles (30 m)^15^, Discovery Bay, Jamaica (55 m)^18^, Moorea, French Polynesia (30 m)^40^, Heron Island, Australia (15–18 m)^41^, and Eilat in Red Sea (45 m)^42,43^.

The similarity between the time series of daily temperature minima at SR (SRmin) and the EASNFS temperature output (mT100, Fig. 6), as well as the correlation between the westward propagating cyclonic/anticyclonic eddies related to cold/warm anomalies of mT100 (Fig. 7), suggests that mesoscale eddies play an important role in shaping the temporal variation of daily temperature minima in deep coral reefs of NW. Mesoscale eddies that have occurred in East Taiwan and the Luzon Strait have been shown to influence the environmental conditions in NW^44,45^. Furthermore, the variation in daily temperature minima can be influenced by cold cyclonic eddies and warm anticyclonic eddies, respectively (Fig. 2a).

The large temperature fluctuations at both shallow and deep reefs in NW were likely a result of cold-water intrusions driven by both cold cyclonic eddies and internal wave-induced upwelling. During the mass coral bleaching events in the northwest Pacific in the summer of 2007^5,6,35^, two major cold eddies affected NW; the first resulted in an SRmean <28 °C, and an ILmean <29 °C (both of which were lower than the local coral bleaching threshold of 29 °C [*in situ* seawater temperature maximum monthly mean (MMM) + 1 °C at IL])^32^ for ~10 days from early June to early July (Fig. 2a); the second cold eddy dropped the SRmean and ILmean to <29 °C during August and September. The high-frequency temperature oscillations, then, decreased temperatures below the MMM. Therefore, the low- and high-frequency cold-water intrusions may act as a buffer to reduce thermal stress, as such, could help mitigate coral bleaching, which is increasing in frequency and intensity as a result of climate change-induced ocean warming^7,12,14–17^.

In contrast to the generally beneficial effects of cold eddies, during the same El Niño event in the summer of 2007^5^, a warm eddy caused both the SRmean and ILmean to exceed the local coral bleaching threshold of 29 °C^32^ for ~5 and ~17 days during late July and early August, respectively (Fig. 2a). The maximum DHD for IL and SR were 10.31 and 4.25 °C-days, and the corresponding peak in DHD and DHW from the NOAA CRW satellite sensor data were 17.89 °C-days and 4.34 °C-weeks, respectively (Fig. 3). DHW and DHD values > 4.0 °C-weeks and >4.0 °C-days, respectively, are known to induce coral bleaching elsewhere^6,7,38^. The combination of this warm eddy and the El Niño were coincidental with a severe mass coral bleaching event (up to 25% shallow-water corals bleached) observed that summer at NW^34,35^. In addition, mass coral bleaching events also occurred at nearby other reefs in southern Taiwan^35^, and Dongsha Atoll in the northern SCS^5^. This suggests that a warm eddy impinging in summer could negatively influence coral reefs by increasing the daily mean temperature by 1–2 °C, thereby diminishing the benefits from daily cold-water intrusions discussed in the previous paragraph. This is unfortunate given that mass coral bleaching events, linked with unusually warm summer seawater temperatures, are a global phenomenon and of increasing concern for the viability of coral reefs^6,7,10,12,38^.

Unsurprisingly, the temperature at our deep reef site (30 m) was different from that of our shallow reef site (5 m) in NW. The notion that temperature-induced bleaching is generally restricted to shallow depths urgently needs to be evaluated by pre- and post-bleaching surveys over broader depth ranges (i.e., encompassing the mesophotic zone, >30 m), coupled with long-term temperature monitoring over depth^46,47^. The large variation in temperature minima observed at NW shows the unique conditions experienced in this region and highlights southern Taiwan as a robust site for future research into the effect of large-scale oceanographic patterns on coral reefs^48^.

The sources of cold-water intrusions may originate from depths of ~100 m and were strongly affected by the westward propagating mesoscale eddies from the Pacific basin. The slow temperature variations in the surrounding region might be a result of the migration of Kuroshio current or impinging of mesoscale eddies (Fig. 6). The impinging mesoscale eddies in the western North Pacific, which could propagate for thousands of kilometers, have been studied for years; they can alter the thickness of the mixed layer^49^ or influence the transport of the Kuroshio current east of Taiwan^28^. The time scale relating to these eddies varies from weeks to months. Westward-moving eddies are common and may connect reef populations along the continental margin^50^.

The eddy impact was clearly revealed in our study by high correlations between the daily temperature minima and residual sea levels (Fig. 8). It might consequently be feasible to reconstruct historical temperature minimum in NW from the long-term coastal sea level records to trace the thermal effects on reef corals. The understanding and forecasting of temperature variation and extremes could help elucidate the causes and processes shaping biophysical phenomenon in coral reefs in the future.

We suggest that mesoscale eddies may, by modifying daily temperature variations over a sustained period, play an important role in coral reef ecology. A deeper understanding of the magnitude and time scale of temperature variations is necessary to comprehend the biophysical relationships governing coral reef ecosystems in NW and other locations, such as southern Great Barrier Reef^24^ and upper Florida Keys^23^, which are also influenced by mesoscale eddies. Mesoscale eddies play an important role in various physical, chemical, and biological processes such as facilitating mass transport, inducing nutrient flux, enhancing primary production, and promoting organism dispersal^51–53^. Pursuing more biophysical studies on the effects of mesoscale eddies may provide a better understanding of the influence of temperature extremes and variability on coral reefs.

## Methods

Observation. Seawater temperature and sea level were recorded simultaneously at IL, and temperature was recorded at SR (Fig. 1), from May 25, 2007 to September 8, 2008, with a temporal recording resolution of 10 min. Measurements were taken from bottom depths of 5 and 30 m for IL and SR, respectively. There were temperature gaps in February 2008 at SR due to logger malfunction. Temperature and depth were measured using HOBO® loggers (accuracy ± 0.2 °C and 1.5 cm, respectively, resolution 0.10 °C and 0.41 cm, respectively, response time <1 s, Onset Computer Co., Bourne, MA, USA), which were deployed and replaced every two months by divers. During replacement of the instruments, data from new and old loggers were overlapped for 10 minutes to account for potential systematic errors; mean sea level variation data were removed. The differences between overlapping temperatures and pressure readings were <0.2 °C and <0.05 decibar (<5 cm), respectively.

Time series of raw temperature data, as well as daily mean, daily maximum, and daily minimum temperatures, were used to analyze temporal variation across hourly, daily, weekly, monthly, and seasonal scales. DHD, summed positive deviations of temperatures exceeding 1 °C above the climatology MMM SST and integrated over past 12 days, was calculated to quantify heat accumulation as follows^7^:

$${\rm{DHD}}=\frac{{\sum }_{{\rm{t}}{\rm{-}}12}^{{\rm{t}}}\,\hat{{\rm{T}}}({\rm{t}})}{{\rm{N}}},$$

$$\hat{{\rm{T}}}({\rm{t}})=\mathop{\sum }\limits_{{\rm{i}}-{\rm{N}}}^{{\rm{i}}}\Delta {\rm{T}}({\rm{i}}),\Delta {\rm{T}}({\rm{i}})=\{\begin{array}{l}{\rm{T}}({\rm{i}})-{\rm{MMM}},{\rm{if}}\,\Delta {\rm{T}}({\rm{i}})\ge 1\,{}^{\circ }\,{\rm{C}}\\ 0,{\rm{if}}\,\Delta {\rm{T}}({\rm{i}}) < 1\,{}^{\circ }\,{\rm{C}}\end{array},{\rm{i}}=1 \sim {\rm{N}}$$

where T(i) is observed temperature; N = 144 for the number of samples over one day with a 10-min data logging interval; N = 1 for daily SST satellite data; MMM = 28.96 °C during 1985–2012 around Nanwan Bay (21.925°N, 120.775°E; based on daily SST satellite observations from https://coralreefwatch.noaa.gov/product/5km/index_5km_composite.php)^38^; $\hat{{\rm{T}}}({\rm{t}})$ = sum of ΔT(i) over a moving window of 1-day length; and DHD is the sum of $\hat{{\rm{T}}}({\rm{t}})$ over a 12-day moving window.

Spectral analysis was used to assess the raw temperature time series data at both sites using the Matlab program ‘pwelch.m’ with a 95% confidence interval applied (ver. R2016). Data lengths used for SR and IL were 254 days (data collected before the logger malfunction) and 473 days (whole record shown in Fig. 2a), respectively. Data sections from both sites overlapped by ~50% and were windowed (43 days) with a Hamming function before spectra for each site were calculated. The degree of freedom was 11.8 and 22 (average = 16.9) for SR and IL, respectively. The Nyquist frequency was 72 cpd. The resolved frequency ranged from 0.034 cpd up to 72 cpd. Due to differences in data length, motions with periods longer than half a month were not well resolved. The residual (detided) sea level variations (resL) were calculated from the observed sea level variations at IL with diurnal (K1, O1) and semi-diurnal (M2, S2) tides removed. The relationship between residual sea level and daily temperature minimum was examined via regression.

Model reanalysis output. EASNFS is an application produced by the U.S. Naval Research Laboratory’s Ocean Nowcast/Forecast System^36^ that was adapted from the Princeton Ocean Model (POM) with modifications to accommodate for extensive data assimilation. The model domain covers the entire East Asian Marginal Seas and part of the western Pacific Ocean, ranging from 17.3°S to 52.2°N and from 99.2°E to 158.2°E. The model’s horizontal resolution is ~1/12° (~9 km at 22°N). There are 41 sigma-z levels, with denser levels in the upper water column to resolve data for the upper ocean. The available model output is presented in the form of a 5-day averaged three-dimensional field. EASNFS was based on surface forcing data from the Navy Operational Global Atmospheric Prediction System (NOGAPS)^54^ and assimilated satellite altimeter data and multi-channel SST (MCSST). A detailed description of the forecasting application and data assimilation can be found in Ko and Wang (2014)^55^. EASNFS has been applied to a wide array of studies, for example, to assess anomalous upwelling in NW^36^, and to examine thermal structures in the SCS^56^. The datasets generated during and/or analysed during the current study are available in the figshare repository 10.6084/m9.figshare.10047377^57^.
